# Natural Antimicrobials and Oral Microorganisms: A Systematic Review on Herbal Interventions for the Eradication of Multispecies Oral Biofilms

**DOI:** 10.3389/fmicb.2015.01529

**Published:** 2016-01-14

**Authors:** Lamprini Karygianni, Ali Al-Ahmad, Aikaterini Argyropoulou, Elmar Hellwig, Annette C. Anderson, Alexios L. Skaltsounis

**Affiliations:** ^1^Department of Operative Dentistry and Periodontology, Center for Dental Medicine, University of FreiburgFreiburg, Germany; ^2^Department of Pharmacognosy and Natural Product Chemistry, Faculty of Pharmacy, National and Kapodistrian University of AthensAthens, Greece

**Keywords:** plant extracts, medicinal herbs, multispecies oral biofilms, antiadhesive and antimicrobial properties, oral diseases

## Abstract

Oral diseases such as caries and periodontitis are mainly caused by microbial biofilms. Antibiotic therapy has reached its limits with regard to antimicrobial resistance, and new therapeutic measures utilizing natural phytochemicals are currently a focus of research. Hence, this systematic review provides a critical presentation of the antimicrobial effects of various medicinal herbs against *in vitro, ex vivo*, and *in situ* formed multispecies oral biofilms. Searches were performed in three English databases (PubMed, EMBASE, CAMbase) and the electronic archives of five German journals from the times of their establishment until October 10th, 2014, with the search terms “(*plant extracts* OR *herbal extracts* OR *plant* OR *herb*) AND (*oral biofilm* OR *dental biofilm* OR *dental plaque* OR *oral disease* OR *dental disease*).” The pooled data were assessed according to Preferred Reporting Items for Systematic Reviews and Meta-Analyses guidelines (PRISMA). Initially, 1848 articles were identified, out of which 585 full-text articles were screened, 149 articles were reevaluated for eligibility and finally, 14 articles met all inclusion criteria. The data of 14 reports disclosed enhanced antiadhesive and antibiofilm activity by the plant extracts obtained from *Vitis vinifera, Pinus* spp., *Coffea canephora, Camellia sinensis, Vaccinium macrocarpon, Galla chinensis, Caesalpinia ferrea* Martius, *Psidium cattleianum*, representative Brazilian plants and manuka honey. Overall, a positive correlation was revealed between herb-based therapies and elimination rates of all types of multispecies oral biofilms. In that context, integrating or even replacing conventional dental therapy protocols with herbal-inspired treatments can allow effective antimicrobial control of oral biofilms and thus, dental diseases.

## Introduction

**Figure 1:**
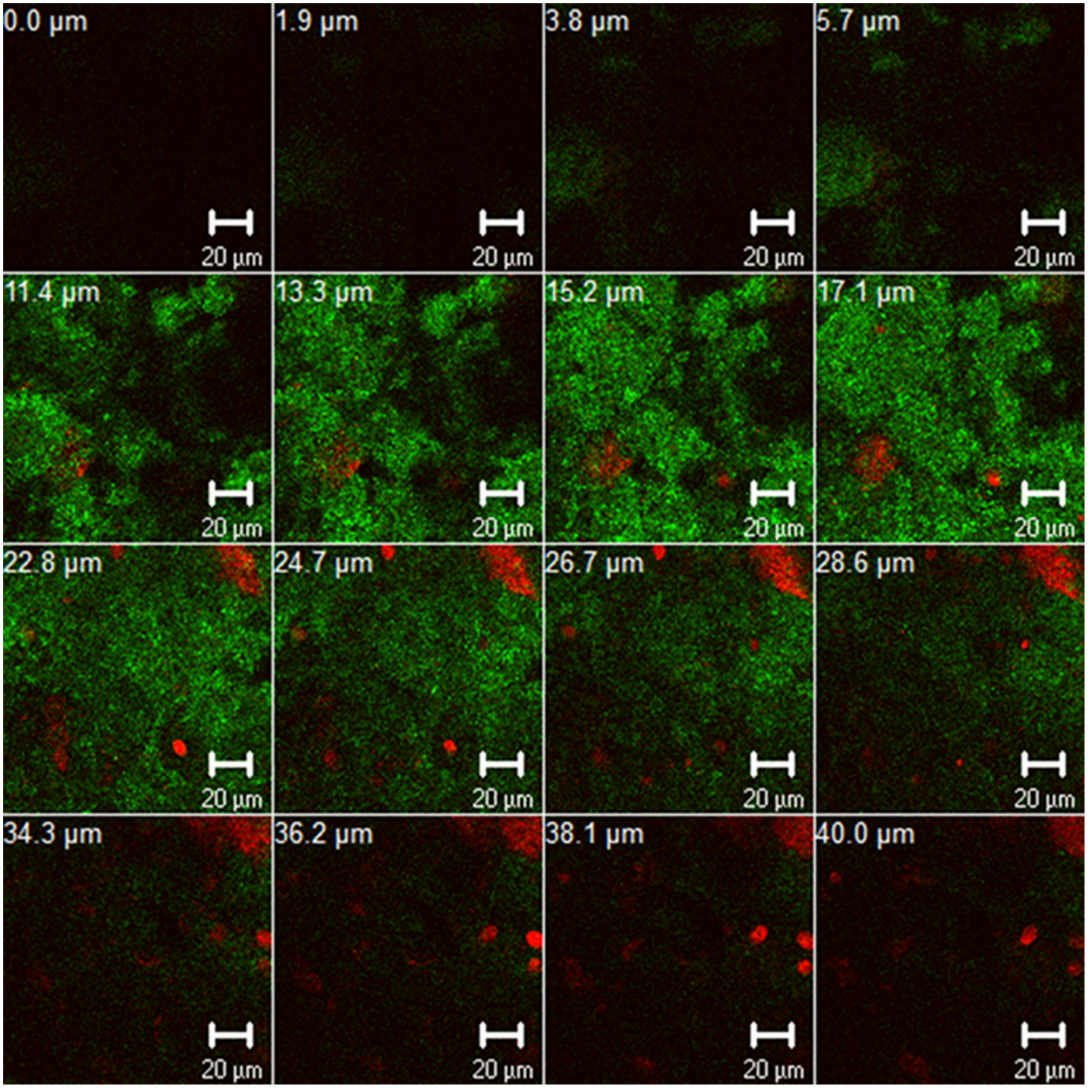
**Z-section gallery of representative confocal laser scanning microscopic (CLSM) image illustrating 3-day old oral biofilm after live/dead staining**. The panel depicts live (green) and dead (red) biofilm microorganisms and contains multiple Z-sections induced by vertical sectioning in 1.9 μm intervals through the oral biofilm above the substratum. Scale bar, 20 μm.

An important trend in microbiological dental research recently is the discovery of new methods to eradicate dental plaque biofilms. It is well-known that the transition of “healthy” oral biofilms into pathological ones is etiologically associated with dental diseases, e.g., caries, periodontitis, or periimplantitis (Madianos et al., 2005). Interestingly, an estimated 700 bacterial species, embedded in extracellular polysaccharide-rich matrix, have been found to dwell within multispecies oral biofilms (Figure 1) (Achtman and Wagner, 2008; Koo et al., 2013; Nikitkova et al., 2013). The “battle” against oral biofilms is a very challenging task, mainly due to their tendency to persist in spite of treatment. This tendency has been attributed to numerous cell-cell communication pathways such as quorum-sensing, horizontal gene transfer, and intrabiofilm metabolic transaction (Kolenbrander et al., 2010). Consequently, biofilm microorganisms can be up to 1000 times more resistant than planktonic bacteria to conventional antimicrobial therapies with antibacterial agents such as antibiotics or chlorhexidine (Welin-Neilands and Svensäter, 2007; Karygianni et al., 2014b). Meanwhile, the ineffectiveness of antibiotics against several microorganisms e.g., methicillin-resistant *Staphylococcus aureus* (MRSA) and vancomycin-resistant enterococci (VRE) is a growing threat in the field of oral health (Smith et al., 2013). The recovery of genes associated with antibiotic resistance to erythromycin, tetracycline, and beta-lactamase from infected root canals also underlines the therapeutic barriers of antimicrobial treatment in dental infections (Rôças and Siqueira, 2013; Al-Ahmad et al., 2014). Finally, the presence of intra- or inter-individual discrepancies challenges the elimination of oral biofilm communities (Al-Ahmad et al., 2007).

In view of the growing ineffectiveness of conventional oral biofilm eradication, the implementation of novel treatments inspired by nature has lately gained increasing interest. The wise statement of Hippocrates “Nature itself is the best physician” has now been updated to include the beneficial influences from the plant kingdom against biofilm-related dental diseases. To date, numerous natural plant extracts have been screened for antimicrobial activity against planktonic and biofilm microorganisms (Groppo et al., 2008; Ramakrishna et al., 2011; Sofrata et al., 2011; Paddon et al., 2013). However, over 300.000 herbs still need to be examined with regard to their potential for antimicrobial activity (Ngo et al., 2013). Despite the fact that numerous reports on the antimicrobial effectiveness of various medicinal herbs against planktonic microorganisms and monospecies oral biofilms have been published in the literature, a summary of information about their effects on multispecies oral biofilms is not available. Therefore, the aim of the present systematic review was to qualitatively summarize the antimicrobial activity of several medicinal herbs against *in vitro, ex vivo* and *in vivo* formed multispecies oral biofilms. To the best of our knowledge, this is the first systematic review of the application of various natural extracts against multispecies oral biofilms.

## Material and methods

### Search strategy

In order to find suitable articles, the following electronic databases were searched from the times of their establishment until 10th October, 2014: PubMed, EMBASE, CAMbase, and the electronic archives of the journals “Zeitschrift für klassische Homöopathie,” “Erfahrungsheilkunde,” “Zeitschrift für Phytotherapie,” “Allgemeine Homöopathische Zeitung,” and “Pharmazeutische Zeitung online.” The search terms for papers associated with herbal extracts and oral biofilms were divided in two groups: intervention (plant extracts OR herbal extracts OR plant OR herb) and condition (oral biofilm OR dental biofilm OR dental plaque OR oral disease OR dental disease). The groups of terms listed above were matched to give 15 different search term combinations. Furthermore, related reviews were considered as an additional source of literature reports. Subsequently, the resulting literature reports were formatted and imported into a mutual Endnote library for all of the searched databases. After the insertion of all relevant combinations in Endnote, all duplicates were automatically removed by the program.

### Inclusion criteria

Herbal interventions were characterized as plant-derived preparations (extracts or fractions) emanating from roots, bulbs, trunks, leaves, seeds, flowers and fruits that were applied against oral biofilms worldwide. In this review *in vitro, in situ*, and *ex vivo* studies which investigated the impact of the aforementioned preparations on *in vitro, ex vivo*, and *in situ* formed multispecies oral biofilms were included. Among the latter, only the ones which were comprised of at least two microbial species could be defined as multispecies and were studied in this review. Only microbial biofilms which consisting of representative oral microorganisms were reviewed. Studies published in both English and German were taken into consideration.

### Exclusion criteria

Purified compounds and essential oils originating from plants were excluded from this study. Reports omitted from this review included randomized controlled trials (RCTs), as well as all other types of clinical studies. Studies not relating to oral biofilms or referring to monospecies oral biofilms were filtered out of this review. Furthermore, reports on the influence of various plant extracts against planktonic microorganisms, even if they were representative of the oral cavity, were not taken into account. Studies combining herbal interventions and routine pharmacologic therapy, e.g., antibiotics allowed for a co-intervention, were not reviewed.

### Study selection

The primary literature research was performed by one author. Afterwards, the resulting titles and abstracts were reevaluated by two independent authors, who were responsible for discarding reports unrelated to the subject according to the established inclusion and exclusion criteria. After this second screening round, the remaining studies were downloaded as full-text articles and were subsequently assessed for eligibility. If full access to the papers was impossible, these studies were excluded at this time point. Finally, irrelevant full-text articles were removed during the last third screening phase against the aforementioned criteria and the studies included in qualitative synthesis were determined.

### Data organization

A standard document was used to organize the information gained from each study. In particular, comprehensive data about the authors, year of publication, type of study, type of herbal intervention, extract concentration, treatment duration, type of multispecies biofilm, types of oral microorganisms within the biofilm, duration of incubation until biofilm formation, number of tested samples, methodological aspects, major outcomes, and limitations were noted. Additional clarification using terms from the listed final data was obtained by the dental library of the University of Freiburg and all other authors of the review with expertise in relevant scientific fields. To ensure the credibility of the extracted lists, the selected full texts were controlled twice. In cases of incongruity, the data were adjusted to the content of the source reports. Due to the heterogeneity of the selected reports, they were further classified into (a) *in vitro*, (b) *ex vivo*, and (c) *in situ* studies.

### Data quality evaluation

The collected data were evaluated according to the Preferred Reporting Items for Systematic Reviews and Meta-Analyses guidelines (PRISMA; Liberati et al., 2009). The latter constitutes an updated version of the statement about the Quality of Reporting of Meta-analyses standards (QUOROM) developed by the QUOROM group. The PRISMA website (http://www.prisma-statement.org/statement.htm) contains the current form of the PRISMA statement. In particular, PRISMA includes an evidence-based set of practical tools, such as a detailed 27-point checklist and a flowchart which supports the classification of the search strategy, study selection, and data assessment process in four stages. The aim of this quality control tool is to aid in literature screening, data extraction, and management and thus, enhance critical evaluation of a wide spectrum of research projects, e.g., randomized clinical trials and other types of intervention reports. To eliminate incongruity the aforementioned processes (data organization and quality) were finally surveyed by a second independent author.

## Results

### Description of selected reports

**Figure 2:**
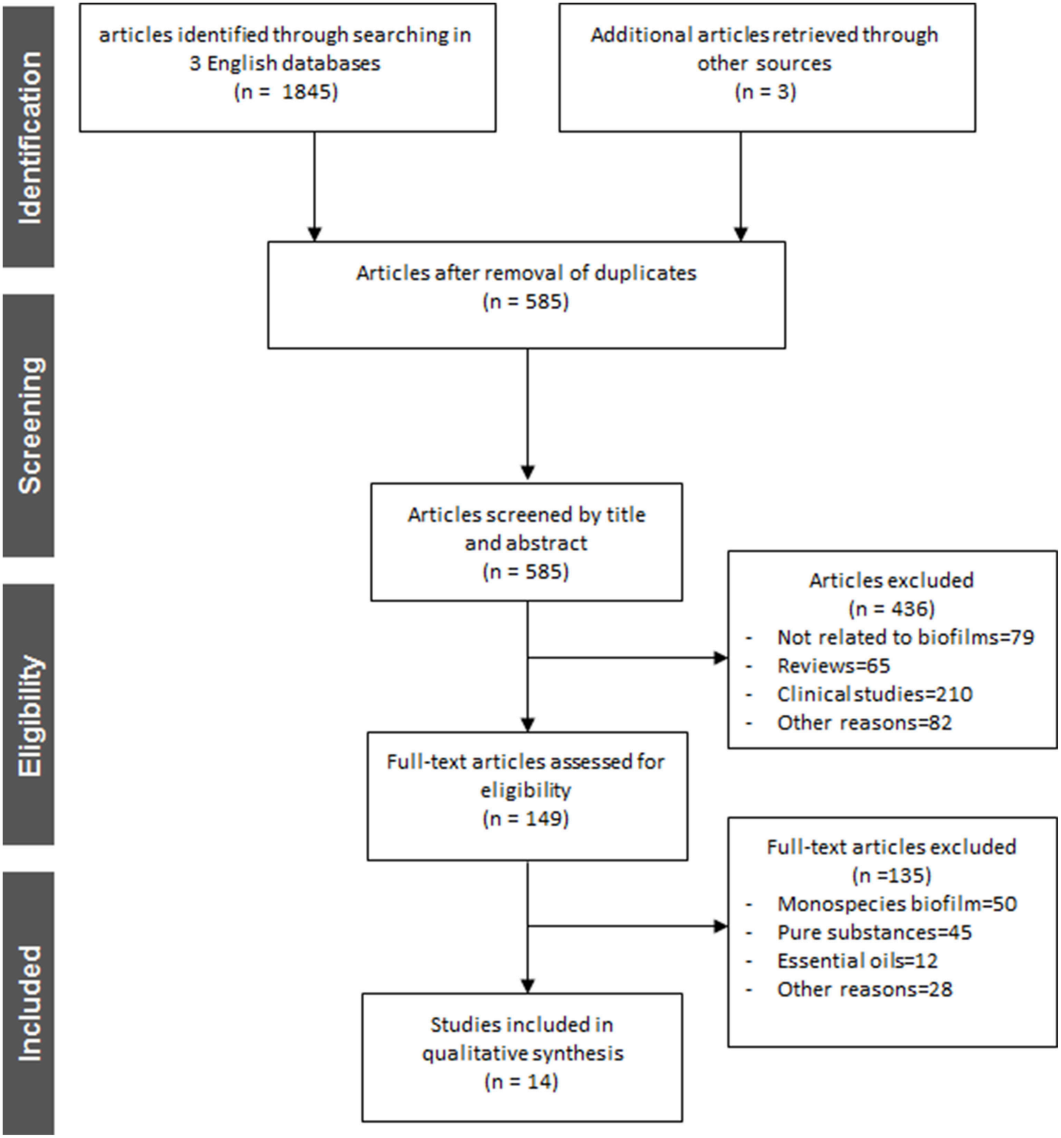
**Flowchart of the search strategy, study selection, and data management procedure**.

Figure 2 depicts an overview of the study selection procedure. Out of a total number of 1848 articles identified after searching in three different English databases and the electronic archives of five German journals, 585 full-text articles were screened upon removal of duplicates. Afterwards, a total of 436 articles that did not meet the inclusion criteria were excluded and 149 full-text articles were reassessed for eligibility. Finally, after this second screening process 135 articles were excluded according to the aforementioned inclusion criteria and 14 of the reports were considered suitable for this review (Yamanaka et al., 2007; Alviano et al., 2008; Furiga et al., 2008, 2014; Hannig et al., 2008, 2009; Xie et al., 2008; Sampaio et al., 2009; Antonio et al., 2011, 2012; Badet and Quero, 2011; Brighenti et al., 2012; Meckelburg et al., 2014; Muñoz-Gonzalez et al., 2014). All 14 selected reports were found in English language databases from the time of their establishment until 10th October, 2014. Further details on each study regarding methodological and outcome aspects are summarized in **Tables 2–4**. All selected studies were written in English and involved treatment of *in vitro, ex vivo* and *in situ* formed multispecies oral biofilms with different plant extracts. The extract treatments were applied with a duration time ranging from 1 min (Furiga et al., 2008, 2014; Hannig et al., 2008, 2009; Xie et al., 2008; Sampaio et al., 2009; Antonio et al., 2011; Brighenti et al., 2012; Meckelburg et al., 2014) to 64 h (Badet and Quero, 2011). In terms of the medicinal herbs, four studies reported the effects of *Vitis* products and by-products, red wine, and/or grape seed extracts on multispecies oral biofilms (Furiga et al., 2008, 2014; Hannig et al., 2009; Muñoz-Gonzalez et al., 2014), three studies highlighted the anti-biofilm properties of *Coffea canephora* extracts (Antonio et al., 2011, 2012; Meckelburg et al., 2014) two studies focused on the antimicrobial traits of tea (black tea, green tea, cistus tea; Hannig et al., 2008, 2009) and one study reported the high antimicrobial efficacy of Chinese galls (Xie et al., 2008) Individual reports on the antibiofilm activity of cranberry juice concentrate (Yamanaka et al., 2007), manuka honey (Badet and Quero, 2011), *Caesalpinia ferrea* Martius fruit extracts (Sampaio et al., 2009), *Psidium cattleianum* leaf extract (Brighenti et al., 2012), and Brazilian plant extracts (Alviano et al., 2008) were also taken into consideration.

To enhance understanding of their great bactericidal potential in the dental field, the most characteristic medicinal herbs with well-established antimicrobial activity against oral microorganisms (planktonic, adherent) are listed below. Table 1 constitutes an overview of the most representative herbs which have already been studied and acknowledged worldwide for their antimicrobial behavior against oral microorganisms.

**Table 1:** **Overview of the representative plant extracts found to have favorable antimicrobial properties against oral bacteria**.

**Name of plant extract**	**Tested oral microorganisms**	**Literature report**
*Azadirachta indica* (Neem sticks)	*Streptococcus* spp.	Wolinsky et al., 1996
*Camellia sinensis* (tea plant)	*Streptococcus mutans, Streptococcus sobrinus*	Hamilton-Miller, 2001; Yoshinaga et al., 2014
*Coffea arabica or canephora* (coffee)	*Streptococcus* spp.	Ferrazzano et al., 2009
*Humulus lupulus* (hop plant)	*Streptococcus* spp.	Shinada et al., 2007
*Vaccinium macrocarpon* (cranberry)	*Streptococcus* spp., *Porphyromonas gingivalis*	Yamanaka et al., 2004, 2007
*Punica granatum* (pomegranate)	*Streptococcus* spp.*, Candida albicans, Porphyromonas gingivalis, Aggregatibacter actinomycetemcomitans, Prevotella intermedia*	de Oliveira et al., 2013; Howell and D'souza, 2013
*Salvadora persica* (miswak)	*Porphyromonas gingivalis, Aggregatibacter actinomycetemcomitans, Haemophilus influenzae*	Sofrata et al., 2007
*Theobroma cacao* (cacao tree)	*Streptococcus mutans, Streptococcus sanguinis*	Percival et al., 2006
*Vitis vinifera* (grape vine)	*Actinomyces oris, Fusobacterium nucleatum, Streptococcus oralis*	Muñoz-Gonzalez et al., 2014
*Myristica fragrans* (nutmeg)	*Streptococcus* spp., *Aggregatibacter actinomycetemcomitans, Porphyromonas gingivalis, Fusobacterium nucleatum*	Shafiei et al., 2012
*Morus alba* (white mulberry)	*Streptococcus mutans*	Islam et al., 2008
*Eucalyptus globulus* (eucalyptus leaves)	*Porphyromonas gingivalis*	Nagata et al., 2006
*Curcuma xanthorrhiza* (Javanese ginger)	*Streptococcus mutans*	Kim et al., 2008
*Arctium lappa* (greater burdock)	*Enterococcus faecalis, Candida albicans*	Pereira et al., 2005
*Mikania laevigata* (guaco)	*Streptococcus* spp.	Yatsuda et al., 2005
*Inula viscosa* (false yellowhead)	*Porphyromonas gingivalis, Streptococcus sobrinus, Enterococcus faecalis*	Karygianni et al., 2014a
*Allium sativum* (garlic)	*Streptococcus* spp., *Enterococcus faecalis, Prevotella intermedia, Fusobacterium nucleatum*	Bakri and Douglas, 2005; Lee et al., 2011
*Hyptis divaricata* (propolis)	*Streptococcus mutans, Lactobacillus* spp.	Dziedzic et al., 2013
*Helichrysum italicum* (curry plant)	*Streptococcus mutans*	Nostro et al., 2004
*Pistacia lentiscus* (mastic gum)	*Porphyromonas gingivalis, Prevotella intermedia, Fusobacterium nucleatum*	Karygianni et al., 2014a
*Aloe vera*	*Streptococcus mutans, Aggregatibacter actinomycetemcomitans, Porphyromonas gingivalis, Bacteroides fragilis*	Fani and Kohanteb, 2012
*Coriandrum sativum* (coriander)	*Candida albicans*	Furletti et al., 2011
*Acacia nilotica* (babul)	mutans streptococci	Gupta and Gupta, 2015
*Hibiscus sabdariffa* (roselle)	*Streptococcus mutans*	Afolabi et al., 2008
*Mangifera indica* (mango)	*Prevotella intermedia, Porphyromonas gingivalis*	Bairy et al., 2002
*Citrus limonum* (lemon)	*Candida albicans, Enterococcus faecalis*	Oliveira et al., 2014
*Cinnamomum zeylanicum* (ceylon cinnamon)	*Actinomyces naeslundii, Prevotella nigrescens, Streptococcus mutans*	Bardají et al., 2015
*Glycyrrhiza uralensis* (Chinese liquorice)	*Streptococcus mutans, Porphyromonas gingivalis*	Villinski et al., 2014
*Hypericum perforatum* (St. John's Wort)	*Streptococcus mutans, Streptococcus sobrinus, Lactobacillus plantarum, Enterococcus faecalis*	Süntar et al., 2015
*Origanum vulgare* (oregano)	*Streptococcus mutans, Candida albicans*	Miller et al., 2015

### Herbal interventions on *In vitro* formed multispecies oral biofilms

Table 2 summarizes the herbal treatments on *in vitro* formed multispecies oral biofilms. A total of seven studies described the effects of several natural extracts on artificial multispecies oral biofilms prepared in the laboratory *in vitro* (Yamanaka et al., 2007; Furiga et al., 2008, 2014; Xie et al., 2008; Sampaio et al., 2009; Badet and Quero, 2011; Muñoz-Gonzalez et al., 2014). The use of honey for treating microbial infections dates back to ancient times. Badet et al. revealed the beneficial effects of manuka honey extract (50–500 μg/ml), a monofloral honey produced in New Zealand and Australia from the nectar of the *Leptospermum scoparium* (Myrtaceae) on biofilms consisting of *Streptococcus mutans, Streptococcus sobrinus, Lactobacillus rhamnosus, Porphyromonas gingivalis*, and *Fusobacterium nucleatum* after incubation for 64 h under aerobic conditions (Badet and Quero, 2011). Furiga et al. published two reports about the antibiofilm activities of various extracts obtained from *Vitis vinifera* (Vitaceae) against artificial oral biofilms mainly composed of *S. mutans, S. sobrinus, L. rhamnosus, P. gingivalis*, and *F. nucleatum* (Furiga et al., 2008, 2014). Apart from wine, which is the primary product derived from *Vitis* plants, research in recent years has focused on the by-products of wine production, such as stems, skins, and seeds, consolidating the observation that agro-industrial wastes are rich in healthy plant ingredients. Red and white grapes originate from the plant *V. vinifera* and are polyphenol-rich sources of hydroxybenzoic acids (ellagic-, gallic-, syringic-, and vanillic acid), hydroxycinnamic acids (ferulic-, p-coumaric-, and caffeic acid), flavonols (catechin, epicatechin, kaempferol, myricetin), stilbenes (tyrosol), procyanidins, and anthocyanins (Furiga et al., 2008). Grapes possess antioxidant, antiinflammatory, antitumor, and antimicrobial properties (Bagchi et al., 2000). The latter include among others antiadhesive activities which can be primarily attributed to organic acids (van Loveren et al., 2012). In earlier reports on beneficial antibacterial effects of grapes and wines within the oral cavity, grapes showed high bactericidal activity against several oral microorganisms e.g., *Actinomyces oris, F. nucleatum*, and *Streptococcus oralis* (Furiga et al., 2008; Muñoz-Gonzalez et al., 2014).

**Table 2:** **Overview of the names and concentrations of the medicinal herbs, bacterial characteristics of oral biofilms, treatment duration, and major outcomes of the studies of herbal intervention on ***in vitro*** formed multispecies oral biofilms**.

**Authors**	**Year**	**Name of medicinal herb/concentration**	**Oral biofilm microorganisms**	**Treatment duration**	**Main results**
Badet and Quero, 2011	2011	Manuka honey extract/50–500 μg/ml	*Streptococcus mutans* ATCC 25175* Streptococcus sobrinus* ATCC 33478* Lactobacillus rhamnosus* ATCC 7469* Porphyromonas gingivalis* ATCC 33277 *Fusobacterium nucleatum* ATCC 10953	Incubation for 64 h under aerobic conditions	- Manuka honey showed antiadhesive properties at a concentration of 200 μg/ml - Manuka honey presented antibiofilm activity at a concentration of 500 μg/ml
Furiga et al., 2008	2008	Red grape marc extract (GME) red wine extract (RWE) pine bark extract (PBE)/50–2000 μg/ml	*Streptococcus mutans* ATCC 25175* Streptococcus sobrinus* ATCC 33478* Lactobacillus rhamnosus* ATCC 7469* Porphyromonas gingivalis* ATCC 33277 *Fusobacterium nucleatum* ATCC 10953	Treatments of 1 min each, every 4 h over 16 h	- Potency against bacterial viability: chlorhexidine > RWE = PBE > GME - GME inhibited the glass surface adhesion by *S. mutans* - GME and PBE inhibited bacterial adhesion to hydroxyapatite - PBE inhibited synthesis of glucosyltransferase by 83.9% at 100 μg/ml
Furiga et al., 2014	2014	Grape seed extract (GSE, 2000 μg/ml), 3-pyridinemethanol hydrofluoride (10.2 mg/ml)	*Streptococcus mutans* ATCC 25175* Streptococcus sobrinus* ATCC 33478* Lactobacillus rhamnosus* ATCC 7469* Porphyromonas gingivalis* ATCC 33277 *Fusobacterium nucleatum* ATCC 10953 *Actinomyces viscosus*	Threefold exposure of 1 min, at 4-h intervals, twice a day over 64 h, between treatments biofilms were incubated	- The combination of GSE and fluoride demonstrated the highest antibiofilm effectiveness - GSE and the combination of GSE and fluoride inhibited glucosyltransferase activity and insoluble glucan synthesis by 43.9 and 65.7%, respectively
Muñoz-Gonzalez et al., 2014	2014	Red wine, dealcoholized red wine, red wine extract (1.6 g/l) enriched either with grape seed extract (2.5 g/100 ml) or inactive dry yeast (IDY, 0.4 g/l)	*Actinomyces oris* OMZ 745 * Fusobacterium nucleatum* OMZ 598 *Streptococcus oralis* OMZ 607 *Streptococcus mutans* UA159 *Veillonella dispar* ATCC 17748	Treatments of 2 min each, at 7-h intervals, twice a day over 7 days	- Red wine, dealcoholized red wine, and red wine extract spiked with grape seed extract decreased CFU values of *F. nucleatum* and *S. oralis* - Red wine extract spiked with IDY reduced oral biofilm viability - No degradation of the flavan-3-ol and quercetin precursors was observed.
Sampaio et al., 2009	2009	*Caesalpinia ferrea* Martius fruit extracts/25–100 μg/ml	*Candida albicans* ATCC 36232 * Streptococcus mutans* ATCC 25175* Streptococcus salivarius* ATCC 7073* Streptococcus oralis* ATCC 10557* Lactobacillus casei* ATCC 7469	Treatments of 1 min each, at 16 h and 30 min, and at 40 h and 30 min over 64 h and 30 min	- Complete inhibition of biofilm formation at 10^−5^ microbial dilution - At 10^−4^ microbial dilution the extract inhibited growth of S. *mutans*, and C. albicans by 0.5 ± 0.1, and 0.7 ±0.1 × 10^6^ CFU, respectively
Yamanaka et al., 2007	2007	Cranberry juice concentrate/250 or 500 μg/ml	*Porphyromonas gingivalis Fusobacterium nucleatum*	Incubation for 24 h under anaerobic conditions	- Cranberry extract hindered synergistic biofilm formation - Cranberry extract (10, 100 μg/ml) hindered Arg-gingipain and Lys-gingipain
Xie et al., 2008	2008	*Galla chinensis* extracts (GCE)/4 mg/ml	*Streptococcus sanguis* ATCC10556 *Streptococcus mutans* ATCC25175 *Actinomyces naeslundii* WVU 627* Lactobacillus rhamnosus* AC 413	Eight treatments of 1 min each, every 12 h over 5 days	- Higher planktonic phase pH values - Lower total viable counts (CFU) after GCE treatment - CFU of *S. sanguis* and *S. mutans* decreased - Fluorescence images of GCE treated biofilms with decreased optical densities

In the first study of the group (Furiga et al., 2008), 1-min applications of red grape marc extract (GME) and red wine extract (RWE) in concentrations ranging from 50 to 2000 μg/ml at 4 h-intervals over 16 h resulted in enhanced antiadhesive effects of GME, while RWE showed the highest antibacterial effects. In the second study of the same group (Furiga et al., 2014), grape seed extract (GSE, 2000 μg/ml), either alone or enriched with 3-pyridinemethanol hydrofluoride, was incubated with biofilms containing the aforementioned microorganisms plus *Actinomyces viscosus* for 1 min, at 4-h intervals over 64 h. The combination of GSE and fluoride exhibited the most antibiofilm properties and decreased glucosyltransferase activity as well as insoluble glucan synthesis by 65.7%. Red wine (dealcoholized or not) and RWE (1.6 g/l) enriched either with grape seed extract (2.5 g/100 ml) or inactive dry yeast (IDY, 0.4 g/l) were also screened against *in vitro* formed multispecies biofilms (*A. oris, F. nucleatum, Streptococcus oralis, S. mutans, Veillonella dispar)* by another group (Furiga et al., 2014). The biofilms were treated for 2 min at 7-h intervals over a period of 7 days. As a result, the total bacterial count of *F. nucleatum* and *S. oralis* were decreased by treatment with red wine and RWE enriched with grape seed. Furiga et al. also studied the effect of a pine bark extract (PBE) against artificial oral biofilms (Furiga et al., 2008). The authors do not mention the pine species used, since PBEs could derive from various species, such as *Pinus pinaster, Pinus maritima, Pinus radiata, Pinus massoniana*. Proanthocyanidins are among the most abundant constituents in these extracts. Antiadhesive effects and high antibacterial potency of PBE were observed with 1-min applications in concentrations ranging from 50 to 2000 μg/ml at 4 h-intervals over 16 h. Interestingly, in the presence of 100 μg/ml PBE glucosyltransferase synthesis decreased by 83.9%. Sampaio et al. (2009) reported complete inhibition of multispecies biofilm formation for *Candida albicans, S. mutans, Streptococcus salivarius, S. oralis*, and *Lactobacillus casei* after 1-min applications over 64 h and 30 min of *C. ferrea* Martius fruit extracts (25–100 μg/ml). *C. ferrea* (syn. *Libidibia ferrea*) Mart. is a leguminous plant found in the north and northeastern semi-arid region of Brazil and is widely used in folk medicine. Tannins are thought to be the major compounds of its fruits extracts, which also contain alkaloids, anthraquinones, sugars, depsides, depsidones, flavonoids, saponins, sesquiterpene lactones, and triterpenes. *Vaccinium* (Ericaceae) is a cosmopolitan genus that contains on the order of 450 species. *Vaccinium macrocarpon* is a native North American fruit. *V. macrocarpon* is the source of cranberries. The cranberry is a fruit rich in polyphenols including proanthocyanidins, anthocyanins, and flavonols (myricetin, quercetin, kaempferol; Duarte et al., 2006). In addition to its significant antioxidant and antitumor effects, cranberry seems to have high antimicrobial efficacy against several Gram-positive and Gram-negative bacteria and fungi known to be involved in recurrent urinary tract infections (Bonifait and Grenier, 2010). Surprisingly, the cranberry is the fruit with the most distinct antiadhesive properties, a fact which highlights its unique ability to strongly inhibit biofilm formation (Yoo et al., 2011). This can be related to various mechanisms such as the decrease of bacterial membrane hydrophobicity (Yamanaka et al., 2004), reduced activity of the enzymes fructosyltransferase and glycosyltransferase (Steinberg et al., 2004) and decreased pH values (Duarte et al., 2006). With regard to oral microorganisms, cranberry was found to be highly effective against oral biofilm members of the genus *Streptococcus* and *P. gingivalis* in previous studies (Yamanaka et al., 2007; Yamanaka-Okada et al., 2008). Yamanaka et al. proved that cranberry extract in concentrations up to 500 μg/ml prevented synergistic biofilm formation by *P. gingivalis* and *F. nucleatum* after 24 h of incubation (Yamanaka et al., 2007).

Finally, the favorable antibiofilm properties of Chinese galls (*Galla Chinensis*) were demonstrated by Xie et al. (2008) on *in vitro* formed biofilms (*Streptococcus sanguis, S. mutans, Actinomyces naeslundii, L. rhamnosus*). Chinese gall is a common traditional Chinese medicine. It is produced through special aphids [Melaphis chinensis (Bell) Baker] parasitized on the leaves or stems of *Rhus chinensis, R. potaninii*, or *R. punjabensis* (Anacardiaceae). Chinese gall has high medicinal value and wide industrial application because of its high levels of gallotannins and various volatile compounds or essential oils. The gallotannins from Chinese galls are quite complex mixtures mostly with degrees of polymerization of 4–11 galloyl units. Biofilm treatment (1 min) with 4 mg/ml *Galla chinensis* extracts (GCE) over 5 days induced increased planktonic phase pH levels and decreased colony forming unit (CFU) values of biofilm microorganisms.

### Herbal interventions on *Ex vivo* formed multispecies oral biofilms

Table 3 summarizes the herbal treatments on *ex vivo* formed multispecies oral biofilms. A total of four studies described the effects of five different natural extracts on multispecies oral biofilms generated *ex vivo* by unstimulated human saliva (Alviano et al., 2008; Antonio et al., 2011, 2012; Meckelburg et al., 2014). Alviano et al. screened four Brazilian plant extracts from *Cocos nucifera, Ziziphus joazeiro, Caesalpinia pyramidalis*, and *Aristolochia cymbifera*, that are used in folk medicine in the northeast of Brazil to treat oral diseases, against oral biofilms from unstimulated human whole mixed saliva (Alviano et al., 2008). After applying 16 mg/ml of each extract onto the biofilms for 30 min, *A. cymbifera, C. pyramidalis* and *C. nucifera* reduced the viable count of adherent microorganisms by 94.2, 72.8, and 64.1% after 48 h of incubation, respectively. The latter two extracts also interfered with the activity of the free radical 1,1-diphenyl-2-picryl-hydrazylhydrate (DPPH). In addition, three further studies by two groups highlighted the antibiofilm activity of *C. canephora* against oral biofilms originating in saliva (Antonio et al., 2011, 2012; Meckelburg et al., 2014). The roasted beans of *Coffea arabica* and *C. canephora* are used for the production of coffee (Nuhu, 2014). Among its active compounds, the alkaloid caffeine (Ferrazzano et al., 2009), chlorogenic acid (Lou et al., 2011), the pyridine alkaloid trigonelline (Antonio et al., 2011), and several diterpenes (bietane, cembrane, guanacastepene A, cafestol) (Bisio et al., 2014) are responsible for the significant antiadhesive behavior of coffee. Coffee was shown to be effective against Gram-positive and Gram-negative microorganisms in an earlier study (Daglia et al., 1998). Within the oral cavity in particular, coffee exhibited antiadhesive activity against *S. mutans* and other oral species of the genus *Streptococcus* (Daglia et al., 2002; Ferrazzano et al., 2009). This could be explained by the ability of caffeine to inhibit quorum sensing, a crucial communication pathway among biofilm microorganisms (Norizan et al., 2013).

**Table 3:** **Overview of the names and concentrations of the medicinal herbs, bacterial characteristics of oral biofilms, treatment duration, and major outcomes of studies on herbal interventions on ***ex vivo*** formed multispecies oral biofilms**.

**Authors**	**Year**	**Name of medicinal herb/concentration**	**Oral biofilm microorganisms**	**Treatment duration**	**Main results**
Alviano et al., 2008	2008	Aqueous extracts from *Cocos nucifera* (husk fiber, coco-cravo), *Ziziphus joazeiro* (innerbark, juazeiro), *Caesalpinia pyramidalis* (leaves, catingueira), and alcoholic extract from *Aristolochia cymbifera* (stem, milhomen)/16 mg/ml	Unstimulated human whole mixed saliva	Treatment for 30 min, incubation under anaerobic conditions for 48 h	- The alcoholic extract of *A. cymbifera* (94.2%) and the aqueous extracts of *C. pyramidalis* (72.8%) and *C. nucifera* (64.1%) reduced the viable count of adherent bacteria - The activity of the free radical 1,1-diphenyl-2-picryl-hydrazyl-hydrate (DPPH) was mostly affected by the aqueous extracts from *C. nucifera* and *C. pyramidalis*
Antonio et al., 2011	2011	Aqueous coffee extract from *Coffea canephora*/50 μg/ml	Unstimulated human whole mixed saliva	Treatments of 1 min each, every 24 h over 7 days	- No significant differences in pH values after treatment with the extract - *C. canephora* aqueous extract induced a 4-log decrease in CFU of *S. mutans* after 3 h treatment
Antonio et al., 2012	2012	Unsweetened and sweetened aqueous coffee extracts from *Coffea canephora*/20 mg/ml	Unstimulated human whole mixed saliva	Treatment for 30 min, incubation under anaerobic conditions for 48 h	- *Coffea canephora* extract extract reduced CFU values in *ex vivo* oral biofilms by 15%. - Sucrose concentrations from 5% up to 20% inhibited growth of biofilm bacteria
Meckelburg et al., 2014	2014	Aqueous coffee extract from *Coffea canephora*/50 μg/ml	Unstimulated human whole mixed saliva	Treatments of 1 min each, every 24 h over 7 days	- Treatment with coffee extract resulted in an increase in calcium concentration after 4 and 7 days - Treatment with coffee extract induced mineral loss from the tooth surfaces up to 30 μm in depth

The first report by Antonio et al. revealed stable pH levels and a 4-log decrease in CFU of *S. mutans* after 1-min incubation of 50 μg/ml aqueous coffee extract from *C. canephora* with oral biofilms originating from unstimulated human saliva over 7 days (Antonio et al., 2011). Interestingly, the results of the second report by the same group were confirmatory (Antonio et al., 2012). In particular, upon treatment for 30 min, 20 mg/ml and sweetened aqueous coffee extracts from *C. canephora* caused a decrease in total bacterial count of 15% in biofilm derived from saliva (Antonio et al., 2012). Similarly, Meckelburg et al. incubated *ex vivo* formed oral biofilms with 50 μg/ml aqueous coffee extract from *C. canephora* for 1 min over 7 days (Meckelburg et al., 2014). In this study, the calcium concentration of the treated biofilms was found to increase, probably due to bacterial lysis.

### Herbal interventions on *In situ* formed multispecies oral biofilms

Table 4 summarizes the herbal treatments on *in situ* formed multispecies oral biofilms. A total of five studies described the effects of 12 different natural extracts on multispecies oral biofilms obtained *in situ* by supragingival dental plaque. *P. cattleianum* commonly known as strawberry guava, Chinese guava, cattley guava, Jeju guava, cherry guava, purple guava, waiawi, guayaba, or araçá is an exotic tropical plant belonging to the Myrtaceae family and native to the Atlantic coast of Brazil. Its leaves are rich in vitamin C and phenolic compounds, including epicatechin and gallic acid as the main components. They also contain flavonoids, saponins, cardiac glycosides, anthraquinones, and tannins. More specifically, Brighenti et al. treated *in situ* oral biofilms with 167 mg/ml *P. cattleianum* leaf extract for 1 min at 12-h intervals over a period of 14 days and reported significantly reduced post-treatment amounts of extracellular polysaccharides and biofilm growth, while pH levels remained stable (Brighenti et al., 2012). Hannig et al. conducted two studies on the antibiofilm effects of various polyphenolic beverages (Hannig et al., 2008, 2009). In their first study, this group tested cistus tea (20 mg/ml) against *in situ* formed initial oral biofilms. Cistus tea is prepared from *Cistus incanus*, a Mediterranean species rich in polymeric polyphenols. A 10-min treatment was followed by further biofilm incubation in the oral cavity for 20, 40, or 109 min (Hannig et al., 2008). In addition to a decrease in peroxidase activity after 40 min, cistus tea reduced the CFU values of the adherent bacteria, whereas glucosyltransferase-, amylase-, and lysozyme activities were not influenced. In the subsequent study from the same group, a variety of polyphenolic beverages (red wine, purple grape juice, cistus tea, black tea, green tea) were screened (Hannig et al., 2009). In brief, 10 mg/ml of each of the aforementioned beverages were applied for 10 min onto *in situ* formed initial oral biofilms and the splint systems were further kept in the oral cavity for either 19 or 109 min. As a result, the total amount of adherent bacteria decreased significantly up to 66%, with cistus tea, red wine, and grape juice showing the highest bactericidal activity. The dried leaves of the plant *Camellia sinensis* produce four well-known varieties of tea, namely black, green, Oolong, and white tea. Polyphenols, especially the flavonoid catechin (30–40% of dry weight), and fluorides are the most active ingredients in tea (Goenka et al., 2013). Tea contains distinct antioxidant properties due to its ability to restrict free radical generation (Reygaert, 2014). Furthermore, tea inhibits the release of antiinflammatory cytokines such as interleukins (IL-6, IL-8, IL-10, IL-12), as well as the receptor activator of nuclear factor kappa-B ligand (RANKL), and acts as an anticancer agent by stimulating apoptotic processes in cancer cells (Chen et al., 2011). In terms of oral health, tea has significant antimicrobial effects. The latter can be attributed to the lysis of the bacterial cell membrane which leads to a reduction in the ability of bacteria to attach to host substrata and form persistent biofilms (Sharma et al., 2012). Other antimicrobial traits involve the interference of tea with the production of fatty acids and enzymes (Reygaert, 2014). For example, tea hinders bacterial energy production by blocking the enzyme ATP synthase (Chinnam et al., 2010). Indeed, numerous studies have revealed a significant bactericidal impact of tea extracts or pure compounds on oral microorganisms such as *Streptococcus* spp., *Aggregatibacter actinomycetemcomitans, Porphyromonas gingivalis, Prevotella intermedia* and *Enterococcus faecalis* (Araghizadeh et al., 2013; Aman et al., 2014).

**Table 4:** **Overview on the names and concentrations of the medicinal herbs, bacterial characteristics of oral biofilms, treatment duration, and major outcomes of the studies on herbal interventions on the ***in situ*** formed multispecies oral biofilms**.

**Authors**	**Year**	**Name of medicinal herb/concentration**	**Oral biofilm microorganisms**	**Treatment duration**	**Main results**
Brighenti et al., 2012	2012	Aqueous extract of *Psidium cattleianum* leaf extract/167 mg/ml	Supragingival dental plaque (palatal appliance)	Treatments of 1 min each, at 12-h intervals, twice a day over 14 days	- The extract inhibited growth of biofilm bacteria - No decrease in pH values after treatment with the extract - The extract reduced the total amount of extracellular polysaccharides
Hannig et al., 2008	2008	Cistus tea/20 mg/ml	Supragingival dental plaque (individual upper jaw splints)	Pellicle formation for 1 min and 15 min, then 10-min treatment, splints kept in the oral cavity for 20 min, 40 min, or 109 min	- Cistus tea reduced the number of detectable adherent bacteria - Cistus tea caused decrease in peroxidase activity after 40 min - Glucosyltransferase, amylase, and lysozyme activities remained unaffected
Hannig et al., 2009	2009	Red wine, purple grape juice, cistus tea, black tea, green tea/10 mg/ml	Supragingival dental plaque (individual upper jaw splints)	Pellicle formation for 1 min, then 10-min treatment, splints kept in the oral cavity for another 19 min or 109 min	- All polyphenolic beverages reduced the number of detectable adherent bacteria - Confocal laser scanning microscopic images showed no discrepancies in the organization of bacterial aggregates - Cistus tea, red wine, and grape juice caused a decrease of up to 66% in oral biofilm bacteria

## Discussion

The present systematic review identified 14 reports on the effects of various medicinal herbs on *in vitro, ex vivo*, and *in situ* formed multispecies oral biofilms. The pooled data from 14 reports revealed the beneficial anti-biofilm behavior of the tested plant extracts (Yamanaka et al., 2007; Alviano et al., 2008; Furiga et al., 2008, 2014; Hannig et al., 2008, 2009; Xie et al., 2008; Sampaio et al., 2009; Antonio et al., 2011, 2012; Badet and Quero, 2011; Brighenti et al., 2012; Meckelburg et al., 2014; Muñoz-Gonzalez et al., 2014). Overall, it can be stated that there is a positive correlation between therapy protocols based on the use of medicinal herbs and the eradication rates of the treated oral biofilms. Independent of the origin of the multispecies oral biofilms (*in vitro, ex vivo*, or *in situ*), all herbs succeeded in reducing the total bacterial counts of the adherent microorganisms. Among the tested plant extracts with confirmed biofilm-killing properties, grape-, pinus-, and other oenological extracts (Furiga et al., 2008, 2014; Hannig et al., 2009; Muñoz-Gonzalez et al., 2014), coffee extracts from *C. canephora* (Antonio et al., 2011, 2012; Meckelburg et al., 2014), as well as polyphenolic beverages such as tea (black tea, green tea, cistus tea; Hannig et al., 2008, 2009) played a major role. Some fruits e.g., cranberry juice concentrate (Yamanaka et al., 2007), *C. ferrea* Martius fruit extracts (Sampaio et al., 2009), manuka honey (Badet and Quero, 2011) and other tree- or leaf extracts such as Chinese galls (Xie et al., 2008), *P. cattleianum* leaf extract (Brighenti et al., 2012), and Brazilian tree extracts (Alviano et al., 2008) also showed pronounced antibiofilm activity.

## Review limitations

For a number of reasons the outcomes from the present review should be interpreted avoiding generalized assumptions. Due to methodological heterogeneity among the studies presented, recommendation of specific treatment protocols with the medicinal herbs studied is not possible. In particular, despite the fact that they are well-described in most reports, the extraction methods applied to the tested herbs, e.g., boiling, differed from one another. Another crucial methodological parameter for the antimicrobial behavior of the herbs studied is the solvent used for the extraction. Here, methanol was utilized as a solvent for the extraction in most reports, whereas aqueous extracts were utilized in some other cases (Alviano et al., 2008; Antonio et al., 2011, 2012; Brighenti et al., 2012; Meckelburg et al., 2014). Furthermore, the quantities used to test the extracts ranged between 50 μg/ml and 300 mg/ml, a fact which can directly affect their anti-biofilm effectiveness. The latter can also be influenced by the duration of treatment, which varied from 1 min (Furiga et al., 2008, 2014; Hannig et al., 2008, 2009; Xie et al., 2008; Sampaio et al., 2009; Antonio et al., 2011; Brighenti et al., 2012; Meckelburg et al., 2014) to 64 h (Badet and Quero, 2011). Obviously, a therapy with an extended treatment time can lead to an overestimation of the positive antimicrobial effects of the plant extract. In addition, the origin of the multispecies oral biofilms studied could be added to the limitations of this review. The different *in vitro, ex vivo* and *in situ* formed multispecies oral biofilms were characterized by a variety of bacterial compositions., which could therefore lead to variation in their response mechanisms toward the applied herbal remedies. The biofilm incubation time prior to and after the herbal treatment, as well as the incubation conditions used (aerobic/anaerobic), also presented discrepancies among the studies presented. Finally, language bias could be a further restriction of this study since only reports written in English were included and only English and German journals were initially taken into consideration.

### Impact of herbs on oral biofilms

In spite of the heterogeneous methodological aspects of the presented studies mentioned above, the general tendency of various natural plant extracts to possess pronounced anti-adhesive and anti-biofilm traits cannot be disputed. Indeed, this antimicrobial behavior was confirmed in the reports analyzed in this review. Since the answer to the general question: “Can herbal intervention eradicate multispecies oral biofilms?” is affirmative, the next question arises: “Exactly which herbal interventions can eradicate multispecies oral biofilms?”

Based on the pooled data in the present review, the extracts from *V. vinifera* seem to be the most promising on the candidate herb list. Interestingly, more than 80% of the polyphenol-rich wine grapes end up in the winemaking process (Thimothe et al., 2007). Thus, the following materials with anti-biofilm properties can be included in this group: red GME, RWE, grape seed extract and red wine. Previous *in vitro* studies also showed a high bactericidal activity for grape extracts against planktonic *S. mutans*, probably due to their interference with glycosyltransferase (GTF) activity, a fact that indirectly inhibits dental plaque accumulation (Thimothe et al., 2007). This assumption was confirmed for multispecies oral biofilms by Furiga et al. (2014), as they showed decreased GTF activity (43.9%) and insoluble glucan synthesis (65.7%) in the presence of grape seed extract. Proanthocyanidins and red pigments are considered to be anti-adhesive components able to modify host and bacterial receptors such as bacterial adhesins (Signoretto et al., 2012).

Furthermore, in the present review coffee was found to be another effective biofilm “killer.” This can be attributed to its enhanced anti-adhesive and anti-biofilm properties as also demonstrated in earlier *in vitro* studies on planktonic bacteria. In particular, low molecular mass substances [chlorogenic acid (Lou et al., 2011), trigonelline (Antonio et al., 2011)] and high molecular mass substances, e.g., melanoidin (Papetti et al., 2007), inhibited the adhesion of *S. mutans* to hydroxyapatite-containing tooth surfaces, as mentioned in the introduction. These components are not only capable of hindering the initiation of the biofilm formation process, but they can also interfere with substantial biofilm communication mechanisms such as quorum sensing (Norizan et al., 2013). However, when combined with additives like sugar, coffee tends to show cariogenic properties within the oral cavity (Anila Namboodiripad and Kori, 2009). Nevertheless, in one of the studies presented (Antonio et al., 2012) sweetened aqueous coffee extracts from *C. canephora* with sucrose concentrations from 5 up to 20% significantly reduced the total bacterial count of biofilm microorganisms.

Tea is currently one of the most popular healthy beverages worldwide. In this review, its beneficial anti-adhesive and anti-biofilm contribution against multispecies oral biofilms was highlighted. As mentioned in the introduction, tea has the ability to either directly affect biofilm viability by destroying the bacterial membrane, or to indirectly affect it by interfering with microbial adhesion to various oral surfaces (Reygaert, 2014). Nevertheless, the consumption of tea with sweeteners should be avoided since the positive impact of tea is then minimized (Morabia and Costanza, 2010). Moreover, the biofilm inhibiting use of fruits, especially cranberries and *C. ferrea* Martius, manuka honey and other tree- or leaf extracts is promising. Several *in vitro* reports have also underlined their favorable impacts on planktonic bacteria (Babu et al., 2012; Eick et al., 2014).

A general response to the question “How do herbal interventions eradicate multispecies oral biofilms?” should include mention of the small-molecule antimicrobial agents (molecular weight, MW < 500) named phytoalexins. These active natural antibiotics consist of heterogeneous components like polyphenols, terpenoids, flavonoids, alkaloids, and glycosteroids. These constitute an herbal “defense reservoir” against pathogenic microorganisms (Hemaiswarya et al., 2008). Interestingly, phytoalexins are capable of acting synergistically in order to eradicate different pathogens. Other plant defense mechanisms against microbial invaders involve the expression of avirulence (Avr) genes, which results in the release of resistance (R) proteins and the local secretion of sugar polymers e.g., callose (Maor and Shirasu, 2005).

Table 5 summarizes the most representative active compounds of the extracts studied in the 14 reviewed reports with antimicrobial activity against oral multispecies biofilms.

**Table 5:** **Representative compounds of extracts with antimicrobial activity against oral multispecies biofilms**.

**Name of medicinal herb**	**Compound**	**Structure**
Red wine extract (RWE)	Catechin	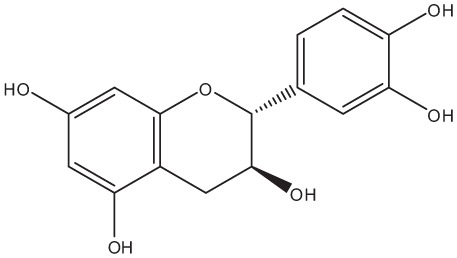
Grape seed extract (GSE)	Catechin	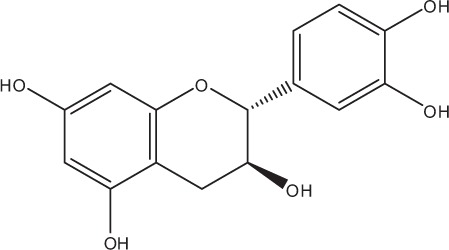
	Epicatechin	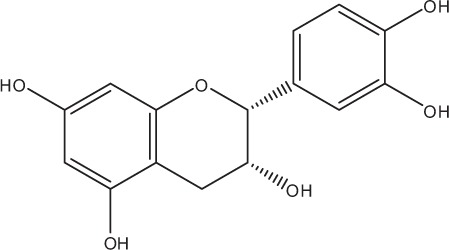
Red wine	Benzoic acids (e.g., protocatechuic acid)	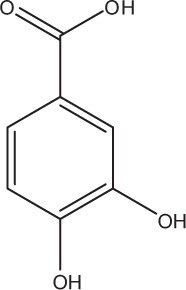
	Phenols (e.g., phloroglucinol)	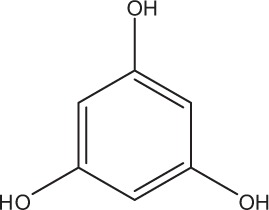
	Cinnamic acids (e.g., p-coumaric acid)	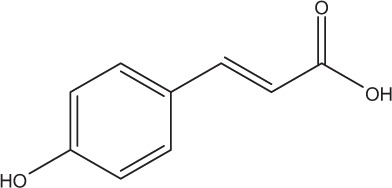
	Stilbene (e.g., resveratrol)	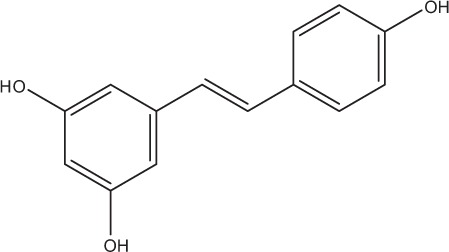
	Procyanidin (e.g., procyanidin B1)	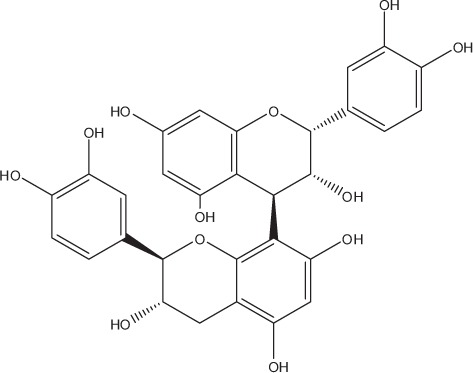
	Flavonols (e.g., quercetin)	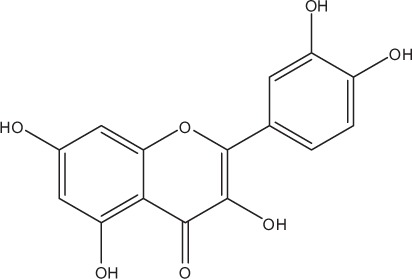
	Anthocyanins (e.g., delphinidin-3-O-glucoside)	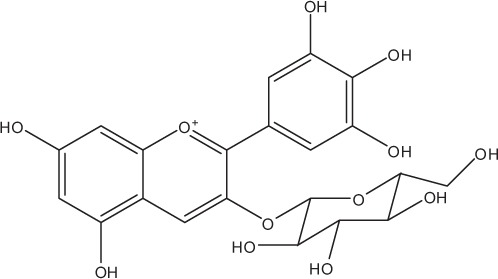
Red wine extract	Benzoic acids (e.g., gallic acid)	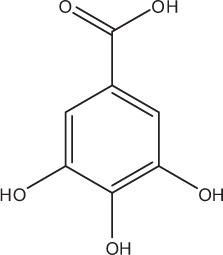
	Phenols (e.g., tyrosol)	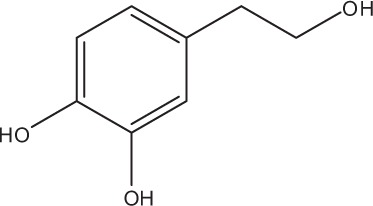
	Cinnamic acids (e.g., coutaric acid)	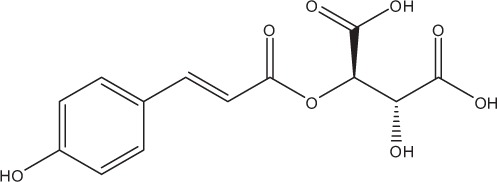
	Stilbene (e.g., resveratrol)	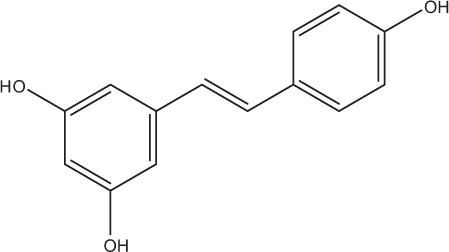
	Procyanidin (e.g., procyanidin B2)	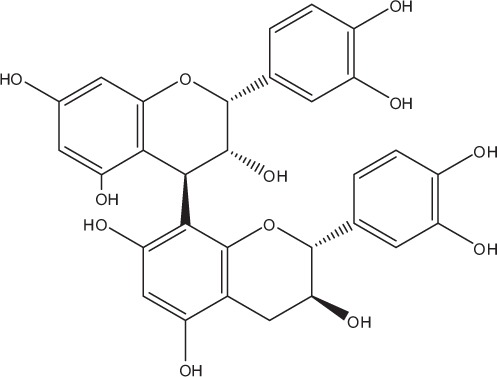
	Flavonols (e.g., myricetin)	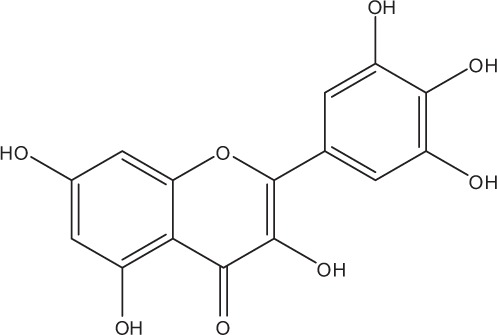
	Anthocyanins (e.g., cyanidin-3-O-glucoside)	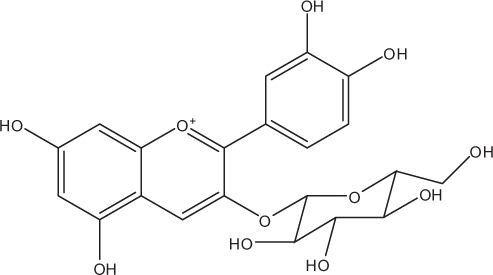
Grape seed extract	Benzoic acids (e.g., gallic acid)	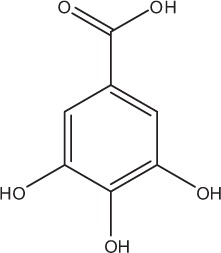
	Procyanidin (e.g., procyanidin B3)	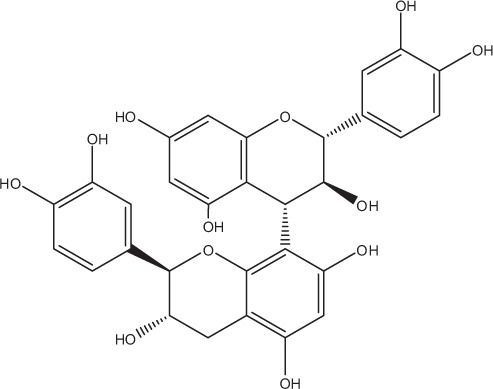
*Galla chinensis* extracts (GCE)	Gallic acid	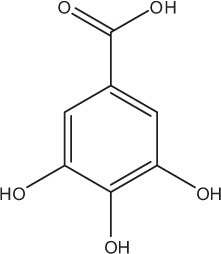
Aqueous coffee extract from *Coffea canephora*	Chlorogenic acid	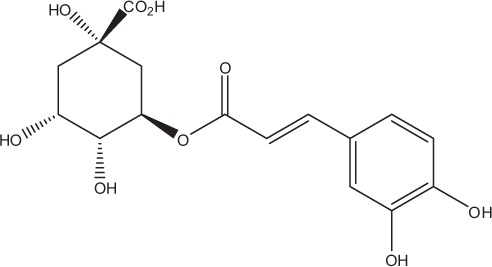
	Neochlorogenic acid	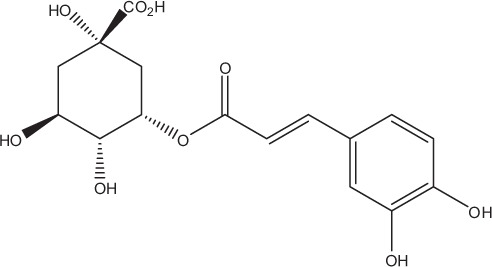
	3-feruloylquinic acid	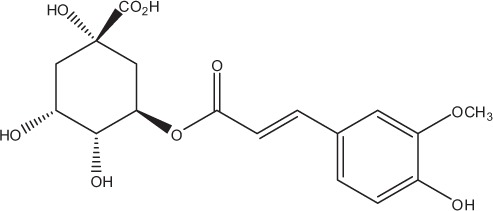
	Cryptochlorogenic acid	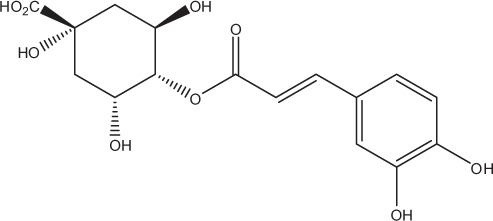
	5-feruloylquinic acid	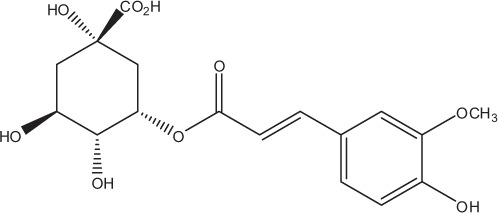
	4-feruloylquinic acid	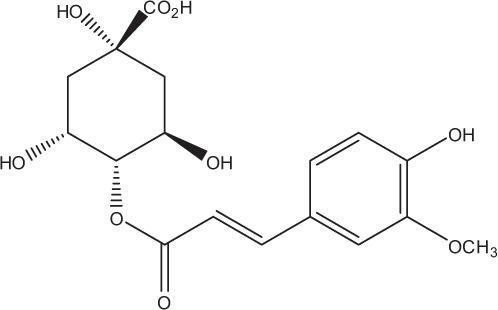
	3,4-dicaffeoylquinic acid	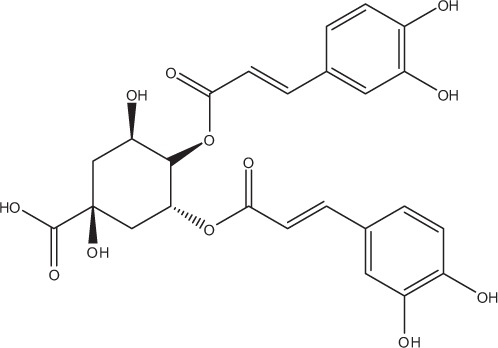
	3,5-dicaffeoylquinic acid	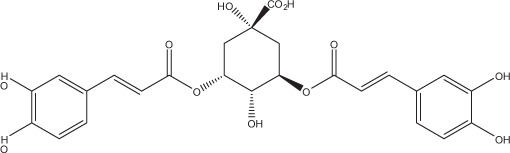
	4,5-dicaffeoylquinic acid	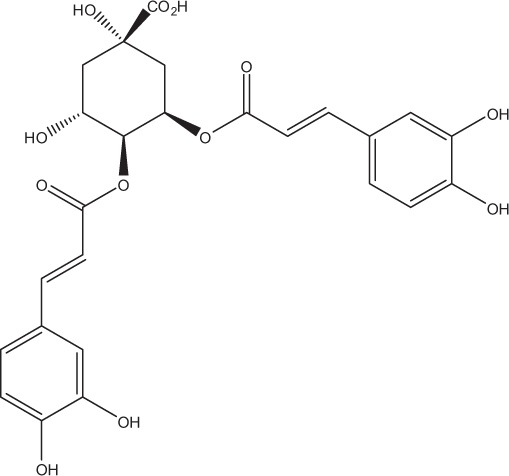
	Caffeic acid	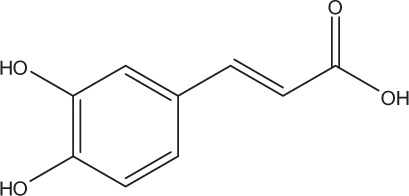
	Caffeine	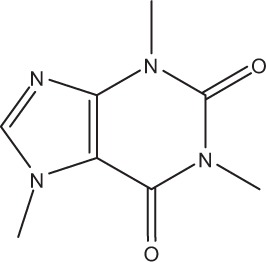
	Trigonelline	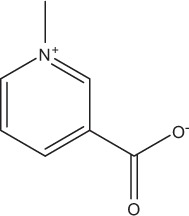
Cistus tea	Catechin	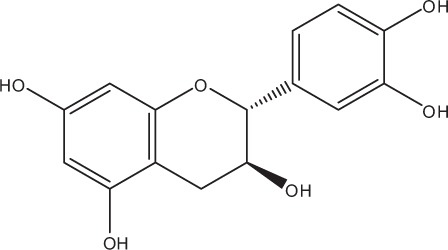
	Myricetin-galactoside	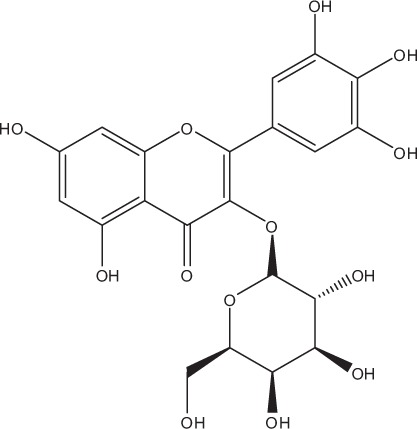
	Myricetin-rhamnoside	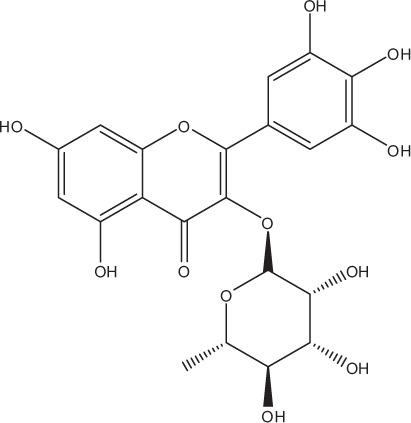
	Quercetin-glucoside	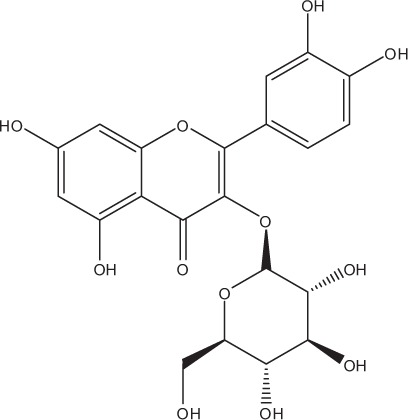

## Future prospects

The following plants have been screened for their antimicrobial activity against planktonic and monospecies biofilm microorganisms and could be also be tested against multispecies biofilm microorganisms in the future.

### *Theobroma cacao* (cacao)

The widely used cacao comes from the plant *Theobroma cacao*. Depending on the degree of cocoa fermentation, the most important flavonoid compounds of cocoa are catechin and epigallocathechin (6–8% of dry weight) (Ferrazzano et al., 2009). In addition to its anti-inflammatory, antioxidant, and anticarcinogenic benefits, cacao possesses significant anticariogenic properties (van Loveren et al., 2012). A possible mechanism for its anticariogenic action involves the inhibition of enzymatic activity of dextransucrase, resulting in reduced production of sucrose-originated extracellular polysaccharides (Ferrazzano et al., 2009). This leads to decreased biofilm formation as shown for *Streptococcus mutans* and *Streptococcus sanguis* in a previous study (Percival et al., 2006).

### *Punica granatum* (pomegranate)

The fruit pomegranate is obtained from the plant *Punica granatum*. Pomegranate demonstrates an astounding antioxidant activity, which can be ascribed to several of its compounds, namely tannins (punicalagins, punicalins), anthocyanins, gallic acid, and ellagic acid (Kote et al., 2011). Pomegranate interferes with biofilm formation by inhibiting antibiotic-resistant bacterial strains and quorum sensing among biofilm microorganisms. Pomegranate was effective at eradicating oral microorganisms e.g., *S. salivarius, S. sanguis, Streptococcus mitis, P. gingivalis, A. actinomycetemcomitans*, and *P. intermedia* in previous studies (Menezes et al., 2006; de Oliveira et al., 2013).

### *Allium sativum* (garlic)

Garlic (*Allium sativum*) is well-known for its antiviral and antimicrobial activities (Shetty et al., 2013). These activities are associated with the alliinase-derived allicin, a bioactive compound found in garlic (Ankri and Mirelman, 1999). Interestingly microorganisms were shown to be up to 1000 times more prone to antibiotic resistance than to allicin resistance (Cutler and Wilson, 2004). Apart from being effective against MRSA, garlic has also demonstrated antibacterial action in the dental field, namely against several planktonic Gram-positive and Gram-negative bacteria (*A. naeslundii, E. faecalis, P. intermedia, P. gingivalis, F. nucleatum)*, as well as against *S. mutans* biofilms (Bakri and Douglas, 2005; Lee et al., 2011).

### *Curcuma xanthorrhiza* (javanese ginger)

Javanese ginger (*Curcuma xanthorrhiza*) contains xanthorrhizol, which shows antiinflammatory, anticancer, and antibacterial activity (Kim et al., 2008). The latter is mainly attributed to the lysis of the bacterial cell membrane by xanthorrhizol (Rukayadi and Hwang, 2006). Javanese ginger was shown to be effective not only against planktonic oral microorganisms, but in earlier studies was also shown to be effective against *S. mutans* biofilms (Rukayadi and Hwang, 2006; Kim et al., 2008).

### *Pistacia lentiscus* (mastic gum)

Mastic gum (*Pistacia lentiscus*) is a resinous exudate with a wide-spectrum of antibacterial and antifungal activity (Sakagami et al., 2009). Mastic gum consists of several volatile compounds, namely α-pinene, β-caryophyllene, β-myrcene, β-pinene, and limonene (Paraschos et al., 2012). Triterpenic acids in particular are considered to be the most active ingredients of mastic gum, with high bactericidal effects, especially against *Helicobacter pylori* (Paraschos et al., 2007). Recently, the effectiveness of mastic gum against anaerobic oral pathogens such as *P. gingivalis, P. intermedia*, and *F. nucleatum* was reported (Karygianni et al., 2014a).

## Conclusions

In conclusion, the outcomes of this review have disclosed the anti-adhesive and anti-biofilm effectiveness of selected natural plant extracts against multispecies oral biofilms. Overall, extracts from *V. vinifera, Pinus* spp. *C. canephora, C. sinensis, V. macrocarpon, G. chinensis, C. ferrea* Martius, *P. cattleianum*, representative Brazilian plants and manuka honey have proven effective against the tested *in vitro, ex vivo*, and *in situ* formed multispecies oral biofilms. The above-mentioned beneficial properties lead to increased interest in the introduction of natural phytochemicals into the therapeutic repertoire of dentistry. Side-effect free medicinal herbs could in this way supplement or even substitute for conventional antiinfectious agents in the battle against periodontitis and other biofilm-related diseases.

## Author contributions

LK, AS conceived the idea for this review; LK, AA conducted search of the relevant databases and participated in the study design; AA-A and ACA organized the data and evaluated their quality; EH, LK, AA, and AS were involved in the data analysis, wrote, and critically reviewed the manuscript. All authors read and approved the final manuscript.

### Conflict of interest statement

The authors declare that the research was conducted in the absence of any commercial or financial relationships that could be construed as a potential conflict of interest.

## References

[B1] Achtman M. Wagner M. (2008). Microbial diversity and the genetic nature of microbial species. Nat. Rev. Microbiol. 6, 431–440. 10.1038/nrmicro1872 18461076

[B2] Afolabi O. C. Ogunsola F. T. Coker A. O. (2008). Susceptibility of cariogenic *Streptococcus mutans* to extracts of Garcinia kola, Hibiscus sabdariffa, and Solanum americanum. West Afr. J. Med. 27, 230–233. 19469401

[B3] Al-Ahmad A. Ameen H. Pelz K. Karygianni L. Wittmer A. Anderson A. C.. (2014). Antibiotic resistance and capacity for biofilm formation of different bacteria isolated from endodontic infections associated with root-filled teeth. J Endod. 40, 223–230. 10.1016/j.joen.2013.07.023 24461408

[B4] Al-Ahmad A. Wunder A. Auschill T. M. Follo M. Braun G. Hellwig E.. (2007). The *in vivo* dynamics of Streptococcus spp., *Actinomyces naeslundii, Fusobacterium nucleatum* and Veillonella spp. in dental plaque biofilm as analysed by five-colour multiplex fluorescence in situ hybridization. J. Med. Microbiol. 56, 681–687. 10.1099/jmm.0.47094-0 17446294

[B5] Alviano W. S. Alviano D. S. Diniz C. G. Antoniolli A. R. Alviano C. S. Farias L. M.. (2008). *In vitro* antioxidant potential of medicinal plant extracts and their activities against oral bacteria based on Brazilian folk medicine. Arch. Oral Biol. 53, 545–552. 10.1016/j.archoralbio.2007.12.001 18243157

[B6] Aman S. Naim A. Siddiqi R. Naz S. (2014). Antimicrobial polyphenols from small tropical fruits, tea and spice oilseeds. Food Sci. Technol. Int. 20, 241–251. 10.1177/1082013213482476 23703103

[B7] Anila Namboodiripad P. Kori S. (2009). Can coffee prevent caries? J. Conserv. Dent. 12, 17–21. 10.4103/0972-0707.53336 20379435 PMC2848806

[B8] Ankri S. Mirelman D. (1999). Antimicrobial properties of allicin from garlic. Microbes Infect. 1, 125–129. 10.1016/S1286-4579(99)80003-3 10594976

[B9] Antonio A. G. Iorio N. L. Farah A. Netto dos Santos K. R. Maia L. C. (2012). Effect of *Coffea canephora* aqueous extract on microbial counts in *ex vivo* oral biofilms: a case study. Planta Med. 78, 755–760. 10.1055/s-0031-1298435 22532021

[B10] Antonio A. G. Iorio N. L. Pierro V. S. Candreva M. S. Farah A. dos Santos K. R.. (2011). Inhibitory properties of *Coffea canephora* extract against oral bacteria and its effect on demineralisation of deciduous teeth. Arch. Oral Biol. 56, 556–564. 10.1016/j.archoralbio.2010.12.001 21185010

[B11] Araghizadeh A. Kohanteb J. Fani M. M. (2013). Inhibitory activity of green tea (*Camellia sinensis*) extract on some clinically isolated cariogenic and periodontopathic bacteria. Med. Princ. Pract. 22, 368–372. 10.1159/000348299 23485656 PMC5586764

[B12] Babu J. Blair C. Jacob S. Itzhak O. (2012). Inhibition of Streptococcus gordonii metabolic activity in biofilm by cranberry juice high-molecular-weight component. J. Biomed. Biotechnol. 2012:590384. 10.1155/2012/590384 22318895 PMC3270421

[B13] Badet C. Quero F. (2011). The *in vitro* effect of manuka honeys on growth and adherence of oral bacteria. Anaerobe 17, 19–22. 10.1016/j.anaerobe.2010.12.007 21195787

[B14] Bagchi D. Bagchi M. Stohs S. J. Das D. K. Ray S. D. Kuszynski C. A.. (2000). Free radicals and grape seed proanthocyanidin extract: importance in human health and disease prevention. Toxicology 148, 187–197. 10.1016/S0300-483X(00)00210-9 10962138

[B15] Bairy I. Reeja S. Siddharth Rao, P. S. Bhat M. Shivananda P.G. (2002). Evaluation of antibacterial activity of *Mangifera indica* on anaerobic dental microglora based on *in vivo* studies. Indian J. Pathol. Microbiol. 45, 307–310. 12785172

[B16] Bakri I. M. Douglas C. W. (2005). Inhibitory effect of garlic extract on oral bacteria. Arch. Oral Biol. 50, 645–651. 10.1016/j.archoralbio.2004.12.002 15892950

[B17] Bardají D. K. Reis E. B. Medeiros T. C. Lucarini R. Crotti A. E. Martins C. H. (2015). Antibacterial activity of commercially available plant-derived essential oils against oral pathogenic bacteria. Nat. Prod. Res. [Epub ahead of print]. 10.1080/14786419.2015.1043630. 26165725

[B18] Bisio A. Schito A. M. Ebrahimi S. N. Hamburger M. Mele G. Piatti G.. (2014). Antibacterial compounds from *Salvia adenophora* Fernald (Lamiaceae). Phytochemistry 110, 120–132. 10.1016/j.phytochem.2014.10.033 25435172

[B19] Bonifait L. Grenier D. (2010). Cranberry polyphenols: potential benefits for dental caries and periodontal disease. J. Can. Dent. Assoc. 76:a130. 20943032

[B20] Brighenti F. L. Gaetti-Jardim E. Jr. Danelon M. Evangelista G. V. Delbem A. C. (2012). Effect of *Psidium cattleianum* leaf extract on enamel demineralisation and dental biofilm composition *in situ*. Arch. Oral Biol. 57, 1034–1040. 10.1016/j.archoralbio.2012.02.009 22386130

[B21] Chen D. Wan S. B. Yang H. Yuan J. Chan T. H. Dou Q. P. (2011). EGCG, green tea polyphenols and their synthetic analogs and prodrugs for human cancer prevention and treatment. Adv. Clin. Chem. 53, 155–177. 10.1016/B978-0-12-385855-9.00007-2 21404918 PMC3304302

[B22] Chinnam N. Dadi P. K. Sabri S. A. Ahmad M. Kabir M. A. Ahmad Z. (2010). Dietary bioflavonoids inhibit *Escherichia coli* ATP synthase in a differential manner. Int. J. Biol. Macromol. 46, 478–486. 10.1016/j.ijbiomac.2010.03.009 20346967 PMC2862773

[B23] Cutler R. R. Wilson P. (2004). Antibacterial activity of a new, stable, aqueous extract of allicin against methicillin-resistant *Staphylococcus aureus*. Br. J. Biomed. Sci. 61, 71–74. 15250668 10.1080/09674845.2004.11732646

[B24] Daglia M. Papetti A. Dacarro C. Gazzani G. (1998). Isolation of an antibacterial component from roasted coffee. J. Pharm. Biomed. Anal. 18, 219–225. 10.1016/S0731-7085(98)00177-0 9863961

[B25] Daglia M. Tarsi R. Papetti A. Grisoli P. Dacarro C. Pruzzo C.. (2002). Antiadhesive effect of green and roasted coffee on *Streptococcus mutans*' adhesive properties on saliva-coated hydroxyapatite beads. J. Agric. Food Chem. 50, 1225–1229. 10.1021/jf010958t 11853508

[B26] de Oliveira J. R. de Castro V. C. das Graças Figueiredo Vilela P. Camargo S. E. Carvalho C. A. Jorge A. O.. (2013). Cytotoxicity of Brazilian plant extracts against oral microorganisms of interest to dentistry. BMC Complement. Altern. Med. 13:208. 10.1186/1472-6882-13-208 23945270 PMC3751599

[B27] Duarte S. Gregoire S. Singh A. P. Vorsa N. Schaich K. Bowen W. H.. (2006). Inhibitory effects of cranberry polyphenols on formation and acidogenicity of *Streptococcus mutans* biofilms. FEMS Microbiol. Lett. 257, 50–56. 10.1111/j.1574-6968.2006.00147.x 16553831

[B28] Dziedzic A. Kubina R. Wojtyczka R. D. Kabala-Dzik A. Tanasiewicz M. Morawiec T. (2013). The antibacterial effect of ethanol extract of polish propolis on mutans streptococci and lactobacilli isolated from saliva. Evid. Based Complement. Alternat. Med. 2013:681891. 10.1155/2013/681891 23606887 PMC3623395

[B29] Eick S. Schäfer G. Kwiecinski J. Atrott J. Henle T. Pfister W. (2014). Honey - a potential agent against *Porphyromonas gingivalis*: an *in vitro* study. BMC Oral Health 14:24. 10.1186/1472-6831-14-24 24666777 PMC3987683

[B30] Fani M. Kohanteb J. (2012). Inhibitory activity of Aloe vera gel on some clinically isolated cariogenic and periodontopathic bacteria. J. Oral Sci. 54, 15–21. 10.2334/josnusd.54.15 22466882

[B31] Ferrazzano G. F. Amato I. Ingenito A. De Natale A. Pollio A. (2009). Anti-cariogenic effects of polyphenols from plant stimulant beverages (cocoa, coffee, tea). Fitoterapia 80, 255–262. 10.1016/j.fitote.2009.04.006 19397954

[B32] Furiga A. Lonvaud-Funel A. Dorignac G. Badet C. (2008). *In vitro* anti-bacterial and anti-adherence effects of natural polyphenolic compounds on oral bacteria. J. Appl. Microbiol. 105, 1470–1476. 10.1111/j.1365-2672.2008.03882.x 18795979

[B33] Furiga A. Roques C. Badet C. (2014). Preventive effects of an original combination of grape seed polyphenols with amine fluoride on dental biofilm formation and oxidative damage by oral bacteria. J. Appl. Microbiol. 116, 761–771. 10.1111/jam.12395 24251548

[B34] Furletti V. F. Teixeira I. P. Obando-Pereda G. Mardegan R. C. Sartoratto A. Figueira G. M.. (2011). Action of *Coriandrum sativum* L. Essential oil upon oral *Candida albicans* biofilm formation. Evid. Based Complement. Alternat. Med. 2011:985832. 10.1155/2011/985832 21660258 PMC3108195

[B35] Goenka P. Sarawgi A. Karun V. Nigam A. G. Dutta S. Marwah N. (2013). *Camellia sinensis* (Tea): implications and role in preventing dental decay. Pharmacogn. Rev. 7, 152–156. 10.4103/0973-7847.120515 24347923 PMC3841993

[B36] Groppo F. C. Bergamaschi Cde C. Cogo K. Franz-Montan M. Motta R. H. de Andrade E. D. (2008). Use of phytotherapy in dentistry. Phytother. Res. 22, 993–998. 10.1002/ptr.2471 18570269

[B37] Gupta D. Gupta R. K. (2015). Investigation of antibacterial efficacy of *Acacia nilotica* against salivary mutans streptococci: a randomized control trial. Gen. Dent. 63, 23–27. 25574715

[B38] Hamilton-Miller J. M. (2001). Anti-cariogenic properties of tea (*Camellia sinensis*). J. Med. Microbiol. 50, 299–302. 10.1099/0022-1317-50-4-299 11289514

[B39] Hannig C. Sorg J. Spitzmüller B. Hannig M. Al-Ahmad A. (2009). Polyphenolic beverages reduce initial bacterial adherence to enamel *in situ*. J. Dent. 37, 560–566. 10.1016/j.jdent.2009.03.017 19394124

[B40] Hannig C. Spitzmüller B. Al-Ahmad A. Hannig M. (2008). Effects of Cistus-tea on bacterial colonization and enzyme activities of the *in situ* pellicle. J. Dent. 36, 540–545. 10.1016/j.jdent.2008.04.002 18468764

[B41] Hemaiswarya S. Kruthiventi A. K. Doble M. (2008). Synergism between natural products and antibiotics against infectious diseases. Phytomedicine 15, 639–652. 10.1016/j.phymed.2008.06.008 18599280

[B42] Howell A. B. D'souza D. H. (2013). The pomegranate: effects on bacteria and viruses that influence human health. Evid. Based Complement. Alternat. Med. 2013:606212. 10.1155/2013/606212 23762148 PMC3671682

[B43] Islam B. Khan S. N. Haque I. Alam M. Mushfiq M. Khan A. U. (2008). Novel anti-adherence activity of mulberry leaves: inhibition of *Streptococcus mutans* biofilm by 1-deoxynojirimycin isolated from Morus alba. J. Antimicrob. Chemother. 62, 751–757. 10.1093/jac/dkn253 18565974

[B44] Karygianni L. Cecere M. Skaltsounis A. L. Argyropoulou A. Hellwig E. Aligiannis N.. (2014a). High-level antimicrobial efficacy of representative Mediterranean natural plant extracts against oral microorganisms. Biomed. Res. Int. 2014:839019. 10.1155/2014/839019 25054150 PMC4098616

[B45] Karygianni L. Ruf S. Follo M. Hellwig E. Bucher M. Anderson A. C.. (2014b). Novel broad-spectrum antimicrobial photoinactivation of *in situ* oral biofilms using visible light plus water-filtered infrared-A (VIS + wIRA). Appl. Environ. Microbiol. 80, 7324–7336. 10.1128/AEM.02490-14 25239897 PMC4249165

[B46] Kim J. E. Kim H. E. Hwang J. K. Lee H. J. Kwon H. K. Kim B. I. (2008). Antibacterial characteristics of *Curcuma xanthorrhiza* extract on *Streptococcus mutans* biofilm. J. Microbiol. 46, 228–232. 10.1007/s12275-007-0167-7 18545974

[B47] Kolenbrander P. E. Palmer R. J. Jr. Periasamy S. Jakubovics N. S. (2010). Oral multispecies biofilm development and the key role of cell-cell distance. Nat. Rev. Microbiol. 8, 471–480. 10.1038/nrmicro2381 20514044

[B48] Koo H. Falsetta M. L. Klein M. I. (2013). The exopolysaccharide matrix: a virulence determinant of cariogenic biofilm. J. Dent. Res. 92, 1065–1073. 10.1177/0022034513504218 24045647 PMC3834652

[B49] Kote S. Kote S. Nagesh L. (2011). Effect of pomegranate juice on dental plaque microorganisms (streptococci and lactobacilli). Anc. Sci. Life 31, 49–51. 23284205 PMC3530267

[B50] Lee H. J. Park H. S. Kim K. H. Kwon T. Y. Hong S. H. (2011). Effect of garlic on bacterial biofilm formation on orthodontic wire. Angle Orthod. 81, 895–900. 10.2319/121010-713.1 21446865 PMC8916176

[B51] Liberati A. Altman D. G. Tetzlaff J. Mulrow C. Gøtzsche P. C. Ioannidis J. P.. (2009). The PRISMA statement for reporting systematic reviews and meta-analyses of studies that evaluate health care interventions: explanation and elaboration. PLoS Med. 6:e1000100. 10.1371/journal.pmed.1000100 19621070 PMC2707010

[B52] Lou Z. Wang H. Zhu S. Ma C. Wang Z. (2011). Antibacterial activity and mechanism of action of chlorogenic acid. J. Food Sci. 76, M398–M403. 10.1111/j.1750-3841.2011.02213.x 22417510

[B53] Madianos P. N. Bobetsis Y. A. Kinane D. F. (2005). Generation of inflammatory stimuli: how bacteria set up inflammatory responses in the gingiva. J. Clin. Periodontol. 32(Suppl. 6), 57–71. 10.1111/j.1600-051X.2005.00821.x 16128830

[B54] Maor R. Shirasu K. (2005). The arms race continues: battle strategies between plants and fungal pathogens. Curr. Opin. Microbiol. 8, 399–404. 10.1016/j.mib.2005.06.008 15996507

[B55] Meckelburg N. Pinto K. C. Farah A. Iorio N. L. Pierro V. S. dos Santos K. R.. (2014). Antibacterial effect of coffee: calcium concentration in a culture containing teeth/biofilm exposed to *Coffea Canephora* aqueous extract. Lett. Appl. Microbiol. 59, 342–347. 10.1111/lam.12281 24909065

[B56] Menezes S. M. Cordeiro L. N. Viana G. S. (2006). *Punica granatum* (pomegranate) extract is active against dental plaque. J. Herb. Pharmacother. 6, 79–92. 10.1080/J157v06n02_07 17182487

[B57] Miller A. B. Cates R. G. Lawrence M. Soria J. A. Espinoza L. V. Martinez J. V.. (2015). The antibacterial and antifungal activity of essential oils extracted from Guatemalan medicinal plants. Pharm. Biol. 53, 548–554. 10.3109/13880209.2014.932391 25332067

[B58] Morabia A. Costanza M. C. (2010). Tea, coffee, and sweet tooth: towards a Japanese paradox. Prev. Med. 50, 157–158. 10.1016/j.ypmed.2010.02.012 20226966

[B59] Muñoz-González I. Thurnheer T. Bartolomé B. Moreno-Arribas M. V. (2014). Red wine and oenological extracts display antimicrobial effects in an oral bacteria biofilm model. J. Agric. Food Chem. 62, 4731–4737. 10.1021/jf501768p 24773294

[B60] Nagata H. Inagaki Y. Yamamoto Y. Maeda K. Kataoka K. Osawa K.. (2006). Inhibitory effects of macrocarpals on the biological activity of *Porphyromonas gingivalis* and other periodontopathic bacteria. Oral Microbiol. Immunol. 21, 159–163. 10.1111/j.1399-302X.2006.00269.x 16626372

[B61] Ngo L. T. Okogun J. I. Folk W. R. (2013). 21st century natural product research and drug development and traditional medicines. Nat. Prod. Rep. 30, 584–592. 10.1039/c3np20120a 23450245 PMC3652390

[B62] Nikitkova A. E. Haase E. M. Scannapieco F. A. (2013). Taking the starch out of oral biofilm formation: molecular basis and functional significance of salivary alpha-amylase binding to oral streptococci. Appl. Environ. Microbiol. 79, 416–423. 10.1128/AEM.02581-12 23144140 PMC3553756

[B63] Norizan S. N. Yin W. F. Chan K. G. (2013). Caffeine as a potential quorum sensing inhibitor. Sensors (Basel) 13, 5117–5129. 10.3390/s130405117 23598500 PMC3673129

[B64] Nostro A. Cannatelli M. A. Crisafi G. Musolino A. D. Procopio F. Alonzo V. (2004). Modifications of hydrophobicity, *in vitro* adherence and cellular aggregation of *Streptococcus mutans* by *Helichrysum italicum* extract. Lett. Appl. Microbiol. 38, 423–427. 10.1111/j.1472-765X.2004.01509.x 15059215

[B65] Nuhu A. A. (2014). Bioactive micronutrients in coffee: recent analytical approaches for characterization and quantification. ISRN Nutr 2014:384230. 10.1155/2014/384230 24967266 PMC4045301

[B66] Oliveira S. A. Zambrana J. R. Iorio F. B. Pereira C. A. Jorge A. O. (2014). The antimicrobial effects of *Citrus limonum* and *Citrus aurantium* essential oils on multi-species biofilms. Braz. Oral Res. 28, 22–27. 10.1590/S1806-83242013005000024 25000605

[B67] Paddon C. J. Westfall P. J. Pitera D. J. Benjamin K. Fisher K. Mcphee D.. (2013). High-level semi-synthetic production of the potent antimalarial artemisinin. Nature 496, 528–532. 10.1038/nature12051 23575629

[B68] Papetti A. Pruzzo C. Daglia M. Grisoli P. Bacciaglia A. Repetto B.. (2007). Effect of barley coffee on the adhesive properties of oral streptococci. J. Agric. Food Chem. 55, 278–284. 10.1021/jf062090i 17227054

[B69] Paraschos S. Magiatis P. Mitakou S. Petraki K. Kalliaropoulos A. Maragkoudakis P.. (2007). *In vitro* and *in vivo* activities of Chios mastic gum extracts and constituents against *Helicobacter pylori*. Antimicrob. Agents Chemother. 51, 551–559. 10.1128/AAC.00642-06 17116667 PMC1797732

[B70] Paraschos S. Mitakou S. Skaltsounis A. L. (2012). Chios gum mastic: a review of its biological activities. Curr. Med. Chem. 19, 2292–2302. 10.2174/092986712800229014 22414110

[B71] Percival R. S. Devine D. A. Duggal M. S. Chartron S. Marsh P. D. (2006). The effect of cocoa polyphenols on the growth, metabolism, and biofilm formation by *Streptococcus mutans* and *Streptococcus sanguinis*. Eur. J. Oral Sci. 114, 343–348. 10.1111/j.1600-0722.2006.00386.x 16911106

[B72] Pereira J. V. Bergamo D. C. Pereira J. O. França Sde C. Pietro R. C. Silva-Sousa Y. T. (2005). Antimicrobial activity of *Arctium lappa* constituents against microorganisms commonly found in endodontic infections. Braz. Dent. J. 16, 192–196. 10.1590/S0103-64402005000300004 16429183

[B73] Ramakrishna Y. Goda H. Baliga M. S. Munshi A. K. (2011). Decreasing cariogenic bacteria with a natural, alternative prevention therapy utilizing phytochemistry (plant extracts). J. Clin. Pediatr. Dent. 36, 55–63. 10.17796/jcpd.36.1.f485870h90174311 22900445

[B74] Reygaert W. C. (2014). The antimicrobial possibilities of green tea. Front. Microbiol. 5:434. 10.3389/fmicb.2014.00434 25191312 PMC4138486

[B75] Rôças I. N. Siqueira J. F. Jr. (2013). Detection of antibiotic resistance genes in samples from acute and chronic endodontic infections and after treatment. Arch. Oral Biol. 58, 1123–1128. 10.1016/j.archoralbio.2013.03.010 23591127

[B76] Rukayadi Y. Hwang J. K. (2006). Effect of coating the wells of a polystyrene microtiter plate with xanthorrhizol on the biofilm formation of *Streptococcus mutans*. J. Basic Microbiol. 46, 410–415. 10.1002/jobm.200510088 17009296

[B77] Sakagami H. Kishino K. Kobayashi M. Hashimoto K. Iida S. Shimetani A.. (2009). Selective antibacterial and apoptosis-modulating activities of mastic. In Vivo 23, 215–223. 19414406

[B78] Sampaio F. C. Pereira Mdo S. Dias C. S. Costa V. C. Conde N. C. Buzalaf M. A. (2009). *In vitro* antimicrobial activity of *Caesalpinia ferrea* Martius fruits against oral pathogens. J. Ethnopharmacol. 124, 289–294. 10.1016/j.jep.2009.04.034 19397986

[B79] Shafiei Z. Shuhairi N. N. Md Fazly Shah Yap N. Harry Sibungkil C. A. Latip J. (2012). Antibacterial activity of Myristica fragrans against oral pathogens. Evid. Based Complement. Alternat. Med. 2012:825362. 10.1155/2012/825362 23049613 PMC3434417

[B80] Sharma A. Gupta S. Sarethy I. P. Dang S. Gabrani R. (2012). Green tea extract: possible mechanism and antibacterial activity on skin pathogens. Food Chem. 135, 672–675. 10.1016/j.foodchem.2012.04.143 22868144

[B81] Shetty S. Thomas B. Shetty V. Bhandary R. Shetty R. M. (2013). An *in-vitro* evaluation of the efficacy of garlic extract as an antimicrobial agent on periodontal pathogens: a microbiological study. Ayu 34, 445–451. 10.4103/0974-8520.127732 24695825 PMC3968712

[B82] Shinada K. Tagashira M. Watanabe H. Sopapornamorn P. Kanayama A. Kanda T.. (2007). Hop bract polyphenols reduced three-day dental plaque regrowth. J. Dent. Res. 86, 848–851. 10.1177/154405910708600908 17720853

[B83] Signoretto C. Canepari P. Stauder M. Vezzulli L. Pruzzo C. (2012). Functional foods and strategies contrasting bacterial adhesion. Curr. Opin. Biotechnol. 23, 160–167. 10.1016/j.copbio.2011.08.006 21906930

[B84] Smith K. Robertson D. P. Lappin D. F. Ramage G. (2013). Commercial mouthwashes are ineffective against oral MRSA biofilms. Oral Surg. Oral Med. Oral Pathol. Oral Radiol. 115, 624–629. 10.1016/j.oooo.2012.12.014 23510687

[B85] Sofrata A. Lingström P. Baljoon M. Gustafsson A. (2007). The effect of miswak extract on plaque pH. An *in vivo* study. Caries Res. 41, 451–454. 10.1159/000107931 17823507

[B86] Sofrata A. Santangelo E. M. Azeem M. Borg-Karlson A. K. Gustafsson A. Pütsep K. (2011). Benzyl isothiocyanate, a major component from the roots of Salvadora persica is highly active against Gram-negative bacteria. PLoS ONE 6:e23045. 10.1371/journal.pone.0023045 21829688 PMC3148225

[B87] Steinberg D. Feldman M. Ofek I. Weiss E. I. (2004). Effect of a high-molecular-weight component of cranberry on constituents of dental biofilm. J. Antimicrob. Chemother. 54, 86–89. 10.1093/jac/dkh254 15163648

[B88] Süntar I. Oyardi O. Akkol E. K. Ozcelik B. (2015). Antimicrobial effect of the extracts from *Hypericum perforatum* against oral bacteria and biofilm formation. Pharm. Biol. [Epub ahead of print]. 10.3109/13880209.2015.1102948. 26510970 PMC11133939

[B89] Thimothe J. Bonsi I. A. Padilla-Zakour O. I. Koo H. (2007). Chemical characterization of red wine grape (*Vitis vinifera* and Vitis interspecific hybrids) and pomace phenolic extracts and their biological activity against *Streptococcus mutans*. J. Agric. Food Chem. 55, 10200–10207. 10.1021/jf0722405 17999462

[B90] van Loveren C. Broukal Z. Oganessian E. (2012). Functional foods/ingredients and dental caries. Eur. J. Nutr. 51(Suppl. 2), S15–S25. 10.1007/s00394-012-0323-7 22535142

[B91] Villinski J. R. Bergeron C. Cannistra J. C. Gloer J. B. Coleman C. M. Ferreira D.. (2014). Pyrano-isoflavans from *Glycyrrhiza uralensis* with antibacterial activity against *Streptococcus mutans* and *Porphyromonas gingivalis*. J. Nat. Prod. 77, 521–526. 10.1021/np400788r 24479468

[B92] Welin-Neilands J. Svensäter G. (2007). Acid tolerance of biofilm cells of *Streptococcus mutans*. Appl. Environ. Microbiol. 73, 5633–5638. 10.1128/AEM.01049-07 17630302 PMC2042095

[B93] Wolinsky L. E. Mania S. Nachnani S. Ling S. (1996). The inhibiting effect of aqueous *Azadirachta indica* (Neem) extract upon bacterial properties influencing *in vitro* plaque formation. J. Dent. Res. 75, 816–822. 10.1177/00220345960750021301 8655780

[B94] Xie Q. Li J. Zhou X. (2008). Anticaries effect of compounds extracted from *Galla chinensis* in a multispecies biofilm model. Oral Microbiol. Immunol. 23, 459–465. 10.1111/j.1399-302X.2008.00450.x 18954351

[B95] Yamanaka A. Kimizuka R. Kato T. Okuda K. (2004). Inhibitory effects of cranberry juice on attachment of oral streptococci and biofilm formation. Oral Microbiol. Immunol. 19, 150–154. 10.1111/j.0902-0055.2004.00130.x 15107065

[B96] Yamanaka A. Kouchi T. Kasai K. Kato T. Ishihara K. Okuda K. (2007). Inhibitory effect of cranberry polyphenol on biofilm formation and cysteine proteases of *Porphyromonas gingivalis*. J. Periodont. Res. 42, 589–592. 10.1111/j.1600-0765.2007.00982.x 17956474

[B97] Yamanaka-Okada A. Sato E. Kouchi T. Kimizuka R. Kato T. Okuda K. (2008). Inhibitory effect of cranberry polyphenol on cariogenic bacteria. Bull. Tokyo Dent. Coll. 49, 107–112. 10.2209/tdcpublication.49.107 19129685

[B98] Yatsuda R. Rosalen P. L. Cury J. A. Murata R. M. Rehder V. L. Melo L. V.. (2005). Effects of Mikania genus plants on growth and cell adherence of mutans streptococci. J. Ethnopharmacol. 97, 183–189. 10.1016/j.jep.2004.09.042 15707750

[B99] Yoo S. Murata R. M. Duarte S. (2011). Antimicrobial traits of tea- and cranberry-derived polyphenols against *Streptococcus mutans*. Caries Res. 45, 327–335. 10.1159/000329181 21720161 PMC3130978

[B100] Yoshinaga Y. Ukai T. Nakatsu S. Kuramoto A. Nagano F. Yoshinaga M.. (2014). Green tea extract inhibits the onset of periodontal destruction in rat experimental periodontitis. J. Periodont. Res. 49, 652–659. 10.1111/jre.12147 25340204

